# Effects of pubic hair grooming on women’s sexual health: a systematic review and meta-analysis

**DOI:** 10.1186/s12905-024-02951-1

**Published:** 2024-03-11

**Authors:** Asmaa Eltobgy, Ahmed Aljabali, Ahmed Farag, Mohammad Elshorbgy, Mona Hamed, Esraa Hamouda, Heba Hamouda, Neveen Refaey, Marwa Kabeel, Sarah Amro, Toka Abouheseba, Mohammed Tarek

**Affiliations:** 1 Department of Obstetrics and Gynecology, Faculty of Medicine For Girls, Al-Azhar University, Cairo, Egypt; 2 Faculty of Medicine, Jordan University of Science and Technology, Irbid, Jordan; 3 Faculty of Medicine, Misr University for Science and Technology, Giza, Egypt; 4 Faculty of Medicine, Gharyan University, Gharyan, Libya; 5 Faculty of Medicine, Menoufia University, Menoufia, Egypt; 6 Department of Physical Therapy for Women’s Health, Faculty of Physical Therapy, Cairo University, Cairo, Egypt; 7 Faculty of Medicine, Kafrelsheikh University, Kafrelsheikh, Cairo, Egypt; 8 Faculty of Medicine, Najah National University, Nablus, Palestine; 9 Faculty of Medicine, Asyut University, Asyut, Egypt; 10 Faculty of Medicine, Al-Azhar University, Cairo, Egypt; 11 Medical Research Group of Egypt (MRGE), Al-Azhar University, P.O. Box 3050, Cairo, Egypt

**Keywords:** Pubic Hair, Grooming, STI, Women's Health, Meta-analysis

## Abstract

**Background:**

Pubic hair grooming involves the partial or complete removal of pubic hair, and it is a common practice among men and women. Grooming is more prevalent in women, who employ various methods such as shaving, waxing and laser removal. However, it is associated with variable rates of post-grooming adverse outcomes including lacerations and sexually transmitted infections (STIs). To the best of our knowledge, this is the first systematic review and meta-analysis comparing women’s sexual health outcomes between those who groom and those who don’t.

**Methods:**

We followed the MOOSE guidelines and conducted a computerized-based search using (PubMed, Web of Science, Scopus, and Ovid Medline), till June 20th, 2022, for eligible studies using the relevant keywords; (pubic hair grooming) OR (pubic hair removal OR Genital hairless OR Bikini hair removal OR pubic hair depilation). Cross-sectional studies included which compared grooming practices among women in terms of motivation and health outcomes. Women’s satisfaction and incidence of STIs were pooled as standardized mean difference (SMD) and odds ratio (OR) respectively.

**Results:**

Twenty-Two cross-sectional studies were included in our review with 73,091 participant.The odds of having gram-negative gonorrheal and chlamydial infection in Pubic hair groomers were found to be statistically significant (OR = 1.55, 95% CI [1.31, 1.84], *P* < 0.001) (OR = 1.56, 95% CI [1.32, 1.85], *P* < 0.001] respectively. There was no difference between groomer and non-groomer women regarding viral infections such as genital herpes (OR = 1.40, 95% CI [0.56, 3.50], *P* = 0.47) and Condyloma acuminata (OR = 1.75, 95% CI [0.51, 6.01], *P* = 0.37). The most common grooming side effect is genital itching (prevalence = 26.9%, *P* < 0.001). Non-electrical razor (prevalence = 69.3%, *P* < 0.001) is the most common grooming method. White women (prevalence = 80.2%, *P* < 0.001) remove pubic hair more frequently compared to black women (prevalence = 12.2%, *P* < 0.001). Women practice complete grooming (50.3%, *P* < 0.001) of the pubic hair more frequently than partial grooming (33.1%, *P* < 0.001). There are no differences in women’s satisfaction between the two groups (SMD = 0.12, 95% CI [-0.16, 0.40], *P* = 0.39).

**Conclusion:**

This review aligns with previous observational studies regarding sexual health outcomes of pubic hair grooming. There is a need to raise awareness among women regarding the safe practice of pubic hair grooming, emphasizing the clarification of hazards and benefits.

**Supplementary Information:**

The online version contains supplementary material available at 10.1186/s12905-024-02951-1.

## Background

Pubic hair grooming, described as partial or complete removal of pubic hair, is considered a prevalent practice in both men and women [1]. Notably, The prevalence of this practice is reportedly high in groomers with more than 80% of women actively engaging in it, as evidenced by multiple studies [1–5].

The primary method employed for pubic hair grooming is shaving, with significantly fewer individuals utilizing wax, electrolysis, laser hair reduction and hair removal cream [6], this grooming behavior is notably influenced by societal trends, as media increasingly promotes the acceptance of new grooming techniques for achieving hairless female genitalia. These trends are intertwined with societal definitions of attractiveness, cleanliness and femininity [3]; therefore, the majority of women remove pubic hair for both sexual and cosmetic reasons [6].

Aesthetic concerns, rather than functional aspects, have been identified as the predominant motivating factor; Surgeons posit that the surge in grooming trends is accountable for this shift, attributing it to heightened visibility of the labia, fostering increased motivation among women to alter their appearance [4]. Concurrently, women are prone to express stronger associations with feelings of cleanliness, comfort, sex appeal, adherence to social norms within their peer group, and affordability as influential factors influencing their chosen pubic hair style [3].

The act of removing pubic hair, specifically, is linked to notions of glamour and heightened sex appeal to a greater degree than the more commonplace removal of underarm or leg hair. Intriguingly, despite these associations, the newly introduced item "it makes me feel clean" emerged as the most widely endorsed aspect classified as 'feminine.' [7]. certainly, motives related to femininity and sexual attractiveness was paramount in the removal of underarm, leg, and pubic hair. Notably, pubic hair removal received a relatively lower rating for femininity but a higher rating for sexual attractiveness compared to other areas. Additionally, self-enhancement motives were more pronounced in individuals who opted for complete pubic hair removal [7]. Moreover, the frequency and extent of pubic hair removal exhibited associations with the consumption of fashion magazines and specific television programs [7]. Predictably, the hair removal industry has evolved into a lucrative multi-million dollar enterprise [8].

In the Middle East, the removal of female pubic hair, rooted in a longstanding tradition of hygiene that spans many centuries and is recommended by Islam, is an integral aspect of cultural practices [9]. According to Islamic religious etiquettes, “initiations of pubic hair removal at menarche and repetition at least once every 40 days are specified”. Notably, a study conducted in Saudi Arabia revealed that 5.5% of participants cited Islam as a reason for pubic hair removal [10]. This aligns with findings from a similar study among Turkish Cypriot women, where 8% reported that Islam recommended pubic hair grooming for religious reasons [9]. Despite contemporary societal trends emphasizing normative pubic hair grooming, these practices, involving adornment, sculpting, and removal, have historical roots spanning centuries and are motivated by a complex interplay of medical, artistic, and cultural considerations [6].

Addressing grooming practices are crucial for health care practitioners, given that these practices represent a cultural norm [11]. Grooming offers potential benefits, such as reducing the risk of pubic lice, but it also presents clinical risks, including genital cuts, irritation, or infection. Furthermore, grooming is recognized as a potential risk factor for some sexually transmitted infections (STIs) [12]. The reported association between grooming and STIs is subject to various confounding factors, including the increased frequency of grooming observed in young women, This demographic is characterized by higher sexual activity, leading to increased exposure to STIs [2]. Additionally the act of grooming pubic hair induces microtrauma in skin's mucocutaneous barrier, facilitating the invasion and spread of pathogens [13]. Notably, a substantial limitation in the current literature on women's pubic hair grooming is its limited generalizability, with many studies relying on convenience samples that exhibit racial and demographic homogeneity. Although some studies have included more diverse populations, they are often constrained to specific geographic regions and limited age ranges [4].

Our main goals in this review to identify, appraise and summarize the evidence from observational studies to understand motivation behind grooming practice of pubic hair among women of different age groups and to examine the extent to which pubic hair removal methods are related to demographic, relational, psychological and sexual characteristics, including female sexual function and STIs.

## Method

### Study registration

The protocol was registered in the PROSPERO database (CRD42022290998). This systematic review was performed according to the MOOSE statement guidelines and all steps were done in a strict adherence to the Cochrane Handbook of Systematic Reviews and Meta-analysis.

### Eligibility criteria and study selection

We considered all observational cross-sectional studies that involved women at different age groups from puberty till menopause practicing pubic hair grooming using different pubic hair removal methods and control group who did not practice pubic hair grooming. The studies needed to report at least one of the following outcomes quantitively or qualitatively; sexually transmitted infections (STIs), adverse events, and post-grooming women’s satisfaction. We excluded studies that did not meet our inclusion criteria. Six independent reviewers independently screened the exported citations in Rayyan QCRI (Qatar Computing Research Institute). We screened studies for eligibility through two sequential steps: Title and Abstract screening for studies matching the inclusion criteria; and Full-text articles of eligible abstracts were retrieved and screened for eligibility to meta-analysis. Conflicts were resolved through discussion, and when consensus couldn’t be reached, a senior reviewer was consulted.

### Search strategy and keywords

We searched PubMed, Cochrane Central, EBSCO, and Scopus, till June 20th, 2022, for eligible studies using the relevant keywords, which were combined to maximize search strategy sensitivity: (pubic hair grooming) OR (pubic hair removal OR Genital hairless OR Bikini hair removal OR pubic hair depilation). Only studies that had been written in the English language without publication date restriction were included. The search was supplemented by scanning all reference lists of retrieved full-text articles. Complete search strategy reported in Supplementary File 1.

### Data extraction

Eight authors extracted relevant data independently from each paper, with equal distribution among each author and collected in extraction tables and online Google sheets. The following data were extracted from each included study: baseline characteristic of the study population (age, race, education, sample size, pubic hair status, frequency of grooming, method of grooming, relationship status, gender of other partner, sexual activity status and sexual frequency), a summary of the design and main findings of included studies (STIs; gonorrhea, chlamydial, genital herpes and condyloma accomunata infection, side effects of grooming such as genital pain, burning, folliculitis, genital itching and women’s satisfaction); and risk of bias domains.

### Quality assessment

Quality was independently assessed by four authors using the Newcastle–Ottawa Scale (NOS), a tool employed for evaluating the quality of observational studies, and was used for the evaluation of cross-sectional studies [14]. This scale uses a “star” system, with maximum of nine stars, to assess the quality of a study in three domains: selection of participants; comparability of study groups; and the ascertainment of interest outcomes. The quality of each study was assessed using the following scoring algorithms: ≥ 7 points were considered as “good”, 4 to 6 points were considered as “fair”, and < 4 points were considered as "poor” quality. Disagreements regarding the quality assessment of the studies were resolved by discussion and consensus.

### Data synthesis

Since all the study outcomes involve dichotomous data from prospectively designed studies, we presented them as Odds ratio (OR) and standardized mean difference (SMD) between the groomers and non-groomers groups. For all outcomes, the OR with the corresponding 95% confidence intervals was pooled in the DerSimonian Liard meta-analysis model using Review Manager Software (version 5.4 for windows). Funnel plots and publication bias tests were generated by Jamovi (version 1.6 for windows) and StatsDirect (version 3.3.5 for windows, professionally licensed to the senior author) [14].

### Statistical analysis

#### Choice of the meta-analysis model

We calculated the pooled effect size for all outcomes according to the DerSimonian Liard meta-analysis model. This random effect model assumes that included studies represent a random sample from the population and assign a slightly higher weight to small studies on the expenses of larger studies. We chose this model because, unlike the fixed-effects model, it accommodates a larger standard error in the pooled estimate, which makes it suitable in case of inconsistent or controversial estimates. Thus, the calculated effects in our meta-analysis are conservative estimates that take into consideration the possible inconsistencies [15].

#### Assessment of heterogeneity

Statistical heterogeneity among studies was evaluated by the Chi-square test (Cochrane Q test).

A chi-square *P* value less than 0.1 were considered as significant heterogeneity. I-square values ≥ 50% were indicative of high heterogeneity [16].

## Results

### Literature search results

**Fig. 1:**
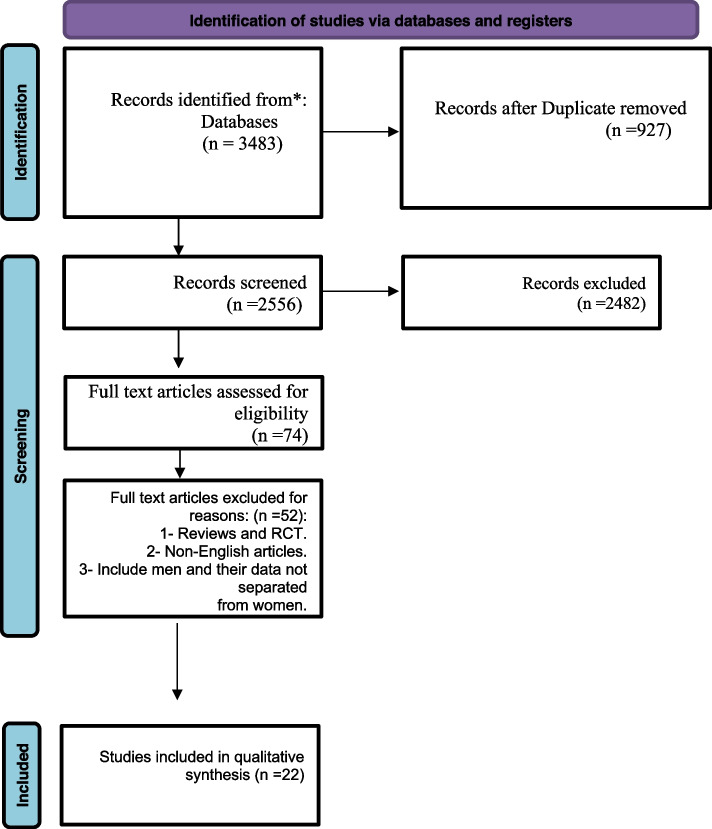
PRISMA flow diagram

Our primary search generated 3483 results. After title and abstract screening, 74 papers were included for full-text screening. The systematic review included 22 out of 74 papers. The references of the listed studies were manually searched, and no more papers were included. The PRISMA flow diagram of the study selection process is presented in Fig. 1.

### Characteristics of the included studies

Twenty-two studies were included in the systematic review with a total of 73,091 women. In all studies, women were categorized as either groomers or non-groomers. A summary of the characteristics of the included studies is provided Table 1. Overall, the risk of bias in the included studies ranged from moderate to low risk, as assessed by the Newcastle Ottawa Quality Assessment Scale (NOS) checklists presented in Table 2. The complete search strategy is reported in Supplementary File 1.

**Table 1:** Characteristics of included studies for SR&MA

#	**Author**	**Year of Publication**	**Study Design**	**Sample size, n**			**Country**	**Recruitment period/year**	**Participants**	**Exposure**	**Main findings**	**Commonest method of grooming**	**Complications of grooming e.g. (STI, Rash, Folliculitis …etc.)**
				**Total**	**Groomer n (%)**	**Non-groomers n (%)**							
1	Toerein et al	2005	Cross-sectional study	678	581 (85.69)	97 (14.31)	UK	_	Female in any age groups who practiced PHR	PHR* using one or more depilatory methods	Significant relationship was observed between younger age and PHR* practices	Shaving (not specified)	_
2	Tiggemann et al	2008	Cross-sectional study	235	194 (74.5)	41 (25.5)	Australia	_	Female of various socioeconomic background aged between 17 and 40 years whom were undergraduate	PHR* using one or more depilatory methods	PHR* has different predictors than the removal of underarm or leg hair due to exposure to media	Shaving (not specified)	_
3	Herbenick et al	2010	Cross-sectional study	2451	1959 (79.9)	492 (20.1)	USA	2008	Women aged 18 to 68 years	PHR* using one or more depilatory methods	Association between total PHR* with younger age, being partnered	Shaving (not specified)	Genital pain
4	Smolak et al	2011	Cross-sectional study	148	96 (65)	52 (35)	USA	_	Women aged above 18 years, undergraduate students	PHR* using one or more depilatory methods	The relationships among normative, sexiness reasons and self-objectification	_	_
5	Bercaw-Pratt et al	2012	Cross-sectional study	171	120 (70.4)	51 (29.6)	USA	_	Adolescents and young women aged 12 to 20 years	PHR* using one or more depilatory methods	PHR* was more common in sexually active participants	Shaving and waxing	_
6	Braun et al	2013	Cross-sectional study	50	47 (94)	3 (6)	New-Zealand	_	Women aged 18 to 48 years, from diverse ethnically; predominantly heterosexual	PHR* using one or more depilatory methods	PHR* were not consistently gendered, With potential impacts on sexual and psychological well-being	_	_
7	Herbenick et al	2013	Cross-sectional study	2453	1410 (57.5)	1043 (42.5)	USA	2013	Women ages 18 to 68 years	PHR* using one or more depilatory methods	Relation between PHR* and their sexual experiences on a day-to-day level with Clinical and educational impact	Non-electric blade razors with shaving cream	Genital pain and irritation
8	DeMariaa et al	2013	Cross-sectional study	1677	1533 (91.4)	144 (8.6)	USA	2010–2011	Women aged 16–40 years whom low-income Hispanic, Black, and White women	PHR* using one or more depilatory methods	PHR* was common among white younger women of varying demographics with under or normal weight, and having 5 or more lifetime sexual partners	Non-electric blade razors and shaving cream	Folliculitis and ingrown hairs
9	DeMaria et al	2014	Cross-sectional study	333	333 (100)	0	USA	2012	Women aged 16 to 40 years	PHR* using one or more depilatory methods	Minor complications commonly occur as a consequence of PHR* and the findings supported the idea of visiting physician to receive health advice on PHR* particularly obese women	Non-electric blade razors	Epidermal abrasions, cuts, bumps, rashes, ingrown hairs, Severe itching and Infection
10	Butler et al	2015	Cross-sectional study	671	644 (96)	27 (4)	USA	_	College women aged above 18 years	PHR* using one or more depilatory methods	Genital grooming and PHR* are common practices among women of college-age. Stronger associations with feelings of cleanliness, comfort, sex appeal, social norms and affordability	Shaving (non-specified)	Genital pain, rash, Itching, cuts
11	Stone et al	2016	Cross-sectional study	126	126 (83.4)	25 (16.6)	_	2015	Women aged above 18 years	PHR* using one or more depilatory methods	Association between PHR* with women being younger, White and were more satisfied with their genitals	_	_
12	Rowen et al	2016	Cross-sectional study	3316	2778 (83.8)	538 (16.2)	USA	2013–2015	Women aged 18 to 65 years	PHR* using one or more depilatory methods	There were demographic differences in grooming practice, which may reflect cultural variations in preference, also no association between grooming, income and relationship status	Non-electric blade razors	_
13	Demaria et al	2016	Cross-sectional study	663	642 (96.8)	21 (3.2)	USA	2013	Young women aged 18–24 years	PHR* using one or more depilatory methods	Education regarding safe PHR* methods is needed, especially for those who initiate pubic hair removal and sexual behaviors concurrently	Non-electric blade razors	Burn, In-grown hairs, severe itching, cuts, rash
14	Sangiorgi et al	2017	Cross-sectional study	52,787	51,386 (97.34)	14,01 (2.65)	Brazil	2015	Women aged above 18 years	PHR* using one or more depilatory methods	Most Brazilian younger women prefer the complete PHR*, especially sexually active women with a stronger preference for complete PHR	Hot wax	_
15	Truesdale et al	2017	Cross-sectional study	3372	3204 (85.3)	168 (14.7)	USA	2014–2017	Women aged 18 to 65 years	PHR* using one or more depilatory methods	Grooming frequency and degree of grooming are independent risk factors for injury	Non-electric blade razors	Lacerations (most common minor injury) serious injuries rare
16	Rouzi et al	2018	Cross-sectional study	400	400 (100)	0	Saudi Arabia	2015–2016	Women aged 16 to 60 years	PHR* using one or more depilatory methods	PHR* common in Saudi women who initiate PHR in early adolescence	Non-electric blade razors	Cuts, bruise, abrasion, severe itching, ingrown hair, rash, burn, allergy, hyperpigmentation
17	Luster et al	2019	Cross-sectional study	214	209 (98.1)	5 (2)	USA	2017–2018	Female university students, at least 18 years of age	PHR* using one or more depilatory methods	PHR* was common among female university students but the findings do not support it as an STI risk factor in this population	Non-electric blade razors	STI (Gonorrhea/chlamydia)
18	obst et al	2019	Cross-sectional study	270	220 (81.6)	50 (18.3)	Australia	2019	Young adult women aged 17 to 25 years	PHR* using one or more depilatory methods	Association between social image and young women decision to engage in behavior that associated with their body image	Shaving (not specified)	_
19	Enzlin et al	2019	Cross-sectional study	1735	1683 (97)	52 (3.1)	Belgium	2011	Women aged 15 to 60 years, they self-identified as heterosexual, bisexual, or homosexual	PHR* using one or more depilatory methods	PHR* is a widespread practice with strongly association with personal, partner-related, sexual, and relational factors	_	Skin rash, itching, bumps
20	Gaither et al	2020	Cross-sectional study	58	54 (93)	4 (7)	USA	2018	Women aged above 18 years received STI testing in the study period, sexually active within the past 3 months	PHR* using one or more depilatory methods	No association between recent grooming and genital STIs	All methods used with no specification	_
21	Beksinska et al	2020	Cross-sectional study	1211	705 (58.2)	506 (41.8)	South Africa	2017–2018	Women aged 16 to 35 years. All were tested for STIs	PHR* using one or more depilatory methods	The practice of PHR* is common among in KwaZulu-Natal, South Africa population with association of high reporting of side effects as injuries.These injuries could put women at a higher risk of STIs	Non-electric blade razors	Itching, pimples, blisters, rash, bleeding, burns and STI (STI (C. trachomatis or N. gonorrhea or HSV2)
22	DeMaria et al	2021	Cross-sectional study	46	46 (100)	0	Italy	2017	Women aged 18 to 45 years	PHR* using one or more depilatory methods	Women engaging in more frequent and earlier waxing PHR* with early onset during adolescence, often upon puberty which influenced by Sexual partners, cultural norms and the desire for cleanliness	Waxing	Burning pain, cuts, ingrown hairs, scarring, swelling, bruising

* abbreviation for Pubic Hair Removal

**Table 2:** Quality assessment of included studies according to Newcastle Ottawa Scale (NOS)

Study ID	Selection (out of 5)				Comparability (out of 2)	outcome (out of 3)		
	1	2	3	4	5	6	7	10
	sample size	Representativeness of the sample	ascertainment of exposure	non-respondents	Comparability based on design or analysis	Assessment of outcome (out of 3)	Reporting the results or statistical test	Total scores (out of 10)
	(* or 0)	(*or 0)	(*or**or0)	(* or 0)	(* or ** or 0)	(* or**or 0)	(* or 0)	
Luster,2019 [2]	0	*	*	*	**	**	0	7
Toerein,2005	0	*	*	0	-	*	0	3
DeMaria,2014 [24]	0	*	*	0	-	*	0	3
Obst,2019 [37]	*	*	*	0	-	*	*	5
Smolak, 2011 [33]	*	*	*	*	**	*	0	7
Tiggemann,2008 [7]	0	0	*	0	0	*	*	3
Bercaw-Pratt,2012 [30]	0	*	0	*	0	*	0	3
Braun-2013 [34]	*	*	**	*	0	**	*	8
DeMaria, 2016 [35]	*	*	*	*	**	**	*	9
Rouzi,2018 [10]	*	*	*	*	*	*	0	6
Sangiorgi, 2017 [36]	*	*	*	*	**	*	*	8
DeMariaa,2013 [6]	0	*	**	*	*	*	*	7
Truesdale,2017 [5]	0	*	**	0	*	*	0	5
Gaither,2020 [39]	*	*	*	0	*	*	*	6
Beksinska,2020 [22]	*	*	*	0	0	**	*	6
Rowen,2016 [4]	*	*	*	*	**	*	*	8
Butler,2015 [3]	*	0	*	0	0	*	*	4
Herbenick,2010 [18]	*	*	*	0	0	**	*	6
Herbenick,2013 [26]	*	*	*	0	0	*	*	5
Enzlin,2019 [38]	*	*	*	*	0	*	*	6
Stone,2016	0	*	**	0	0	**	*	6
DeMaria,2021 [19]	0	*	*	0	0	*	0	3

### Prevalence of pubic hair grooming

**Fig. 2:**
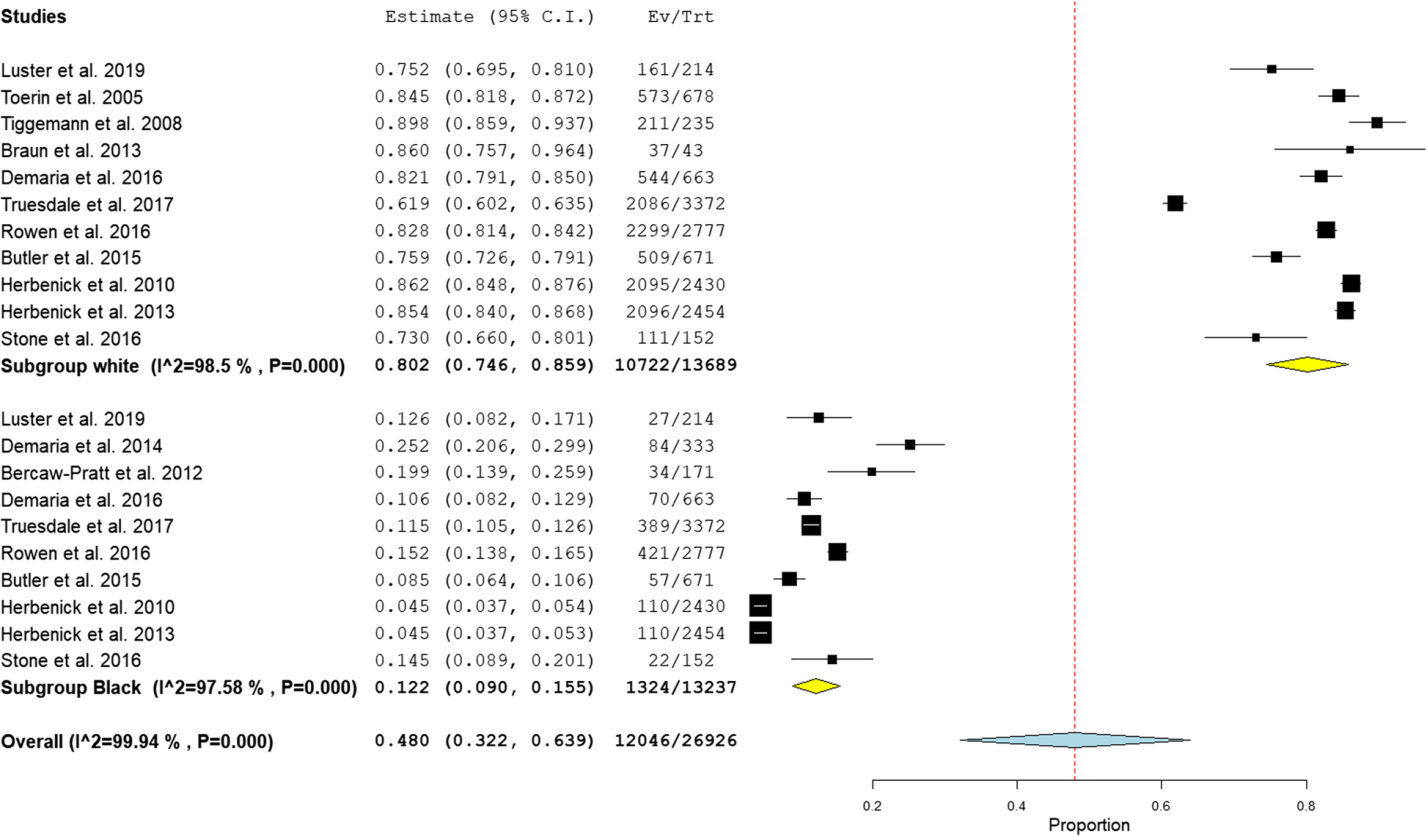
Prevalence of grooming practice in [white and black women]

There was higher prevalence of pubic hair grooming practice among female groomers (93.54%) compared to non-groomers (6.45%). White women (prevalence = 80.2%, *P* < 0.001) remove pubic hair more frequently compared to black women (prevalence = 12.2%, *P* < 0.001), as shown in Fig. 2.

### Grooming types and methods

**Fig. 3:**
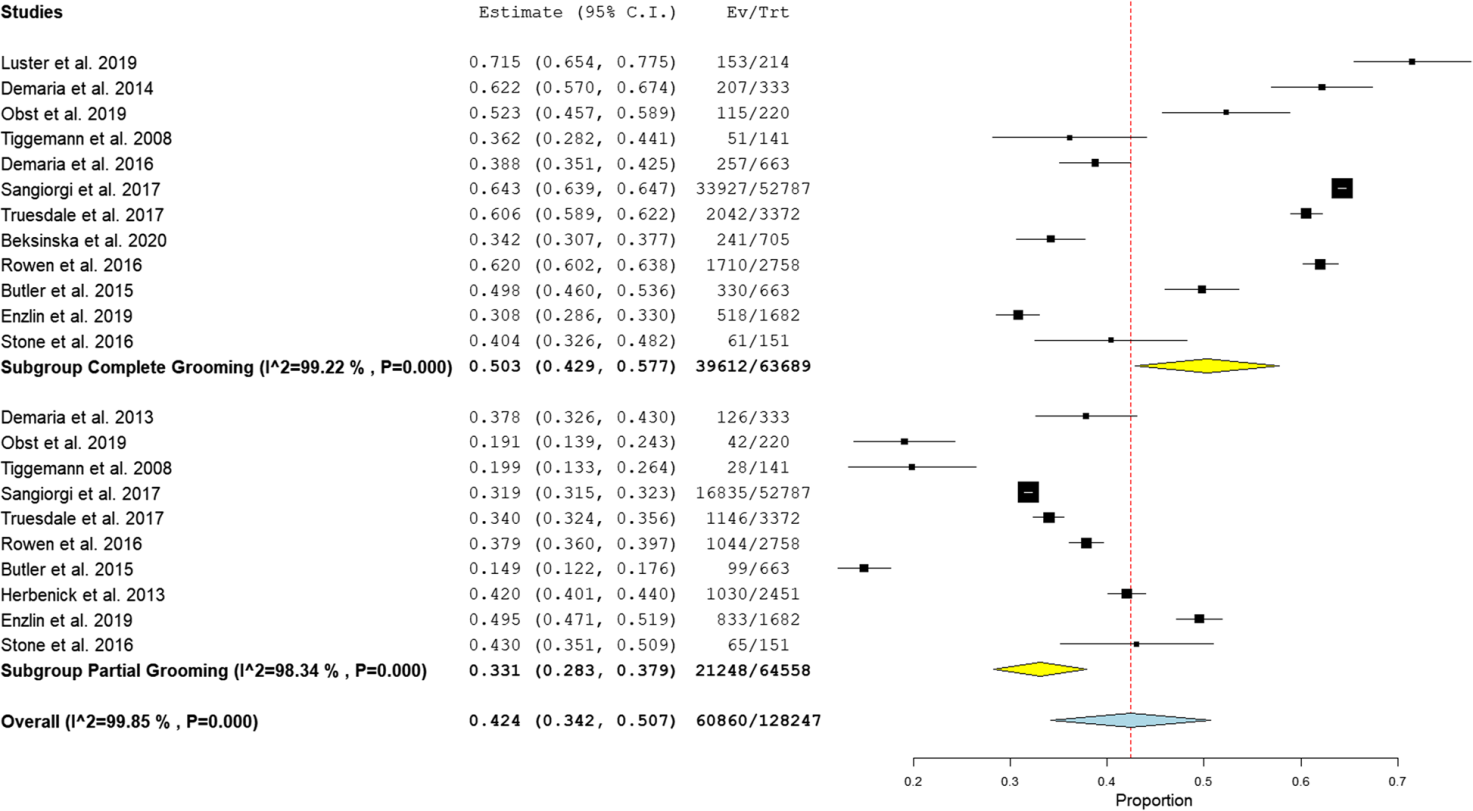
Prevalence of pubic hair grooming types [complete and partial]

**Fig. 4:**
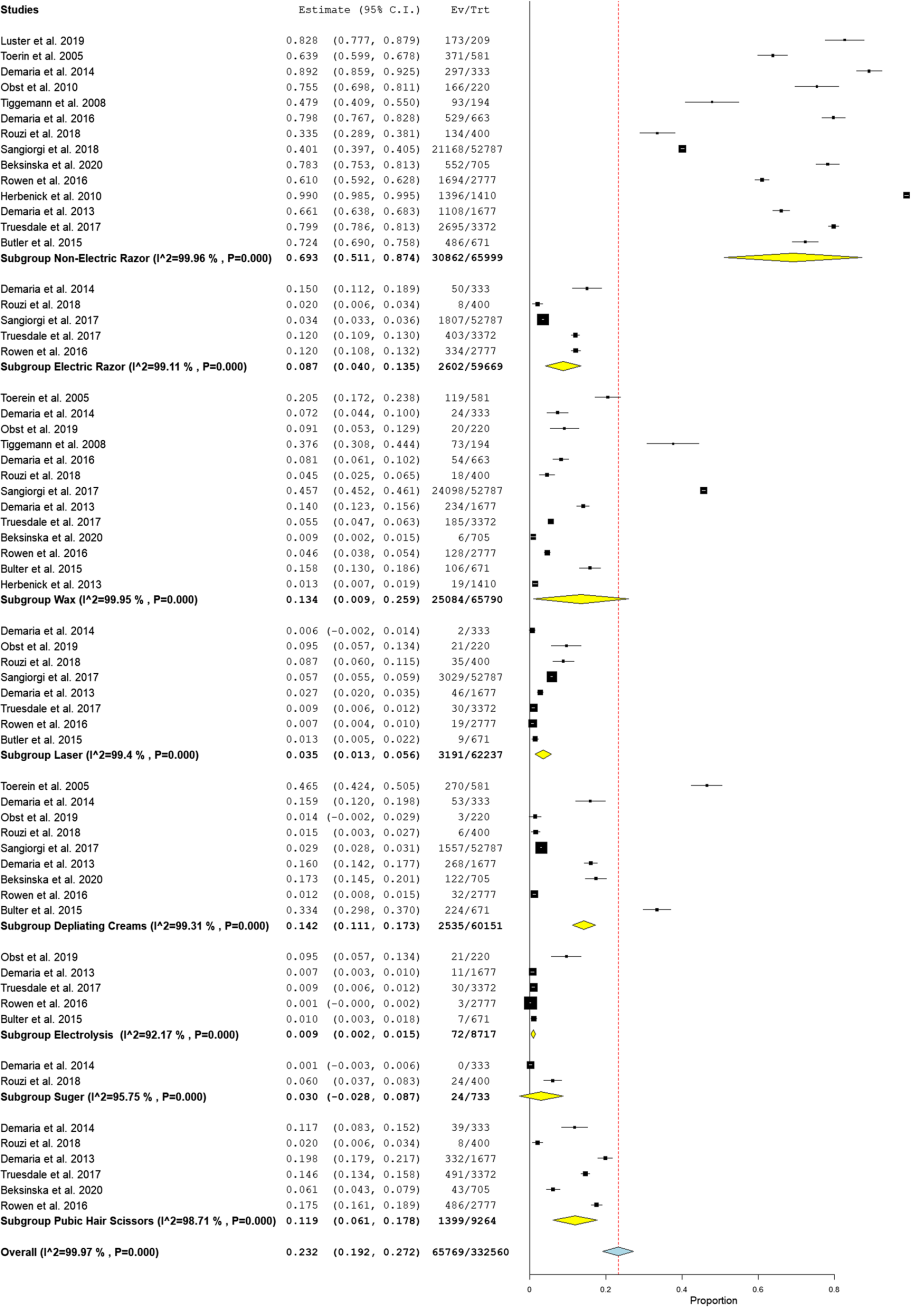
Prevalence of Grooming methods

Pubic hair is more often groomed completely than partially (50.3%, P 0.001) (33.1%, P 0.001) respectively, as shown in Fig. 3. The most common grooming method among women practicing grooming was shaving with a non-electric razor (prevalence = 69.3%, *P* < 0.001), as shown in Fig. 4.

### Grooming satisfaction

**Fig. 5:**
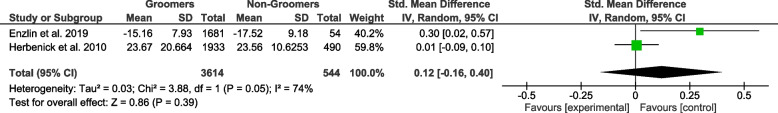
Women grooming satisfaction in [groomers and non-groomers]

Various motivations underlie the practice of pubic hair removal, encompassing considerations such as hygiene, comfort, aesthetic preferences, perceived sex appeal (often linked to being in a partnership), the anticipation of receiving cunnilingus, recent self-observation of one's genital area, and potential social influences, including pressure from family or friends to engage in hair removal practices [10], However there are no differences in women’s satisfaction related to pubic hair removal between groomers and non-groomers (SMD = 0.12, 95% CI [-0.16, 0.40], *P* = 0.39) as shown in Fig. 5.

### Grooming side effect

**Fig. 6:**
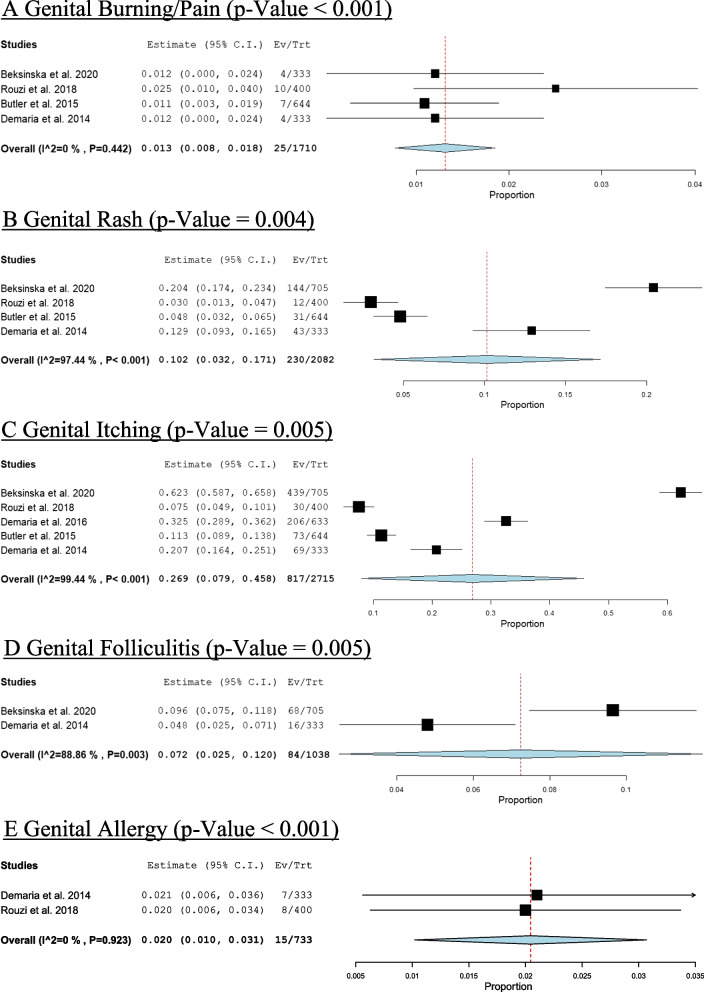
Prevalence of grooming complications. **A** Genital burning/pain. **B** Genital rash. **C** Genital itching. **D** Genital folliculitis. **E** Genital allergy

The most common side effect of grooming was genital itching (prevalence = 26.9%, *P* < 0.001), followed by genital pain and burning (prevalence = 1.3%, *P*>0.001), genital rash (prevalence = 10.2%, *P*>0.001), genital folliculitis (prevalence = 7.2%, *P*>0.001), and genital allergy (prevalence = 2%, *P*>0.001) as shown in Fig. 6.

### Sexually transmitted infections (STIs)

**Fig. 7:**
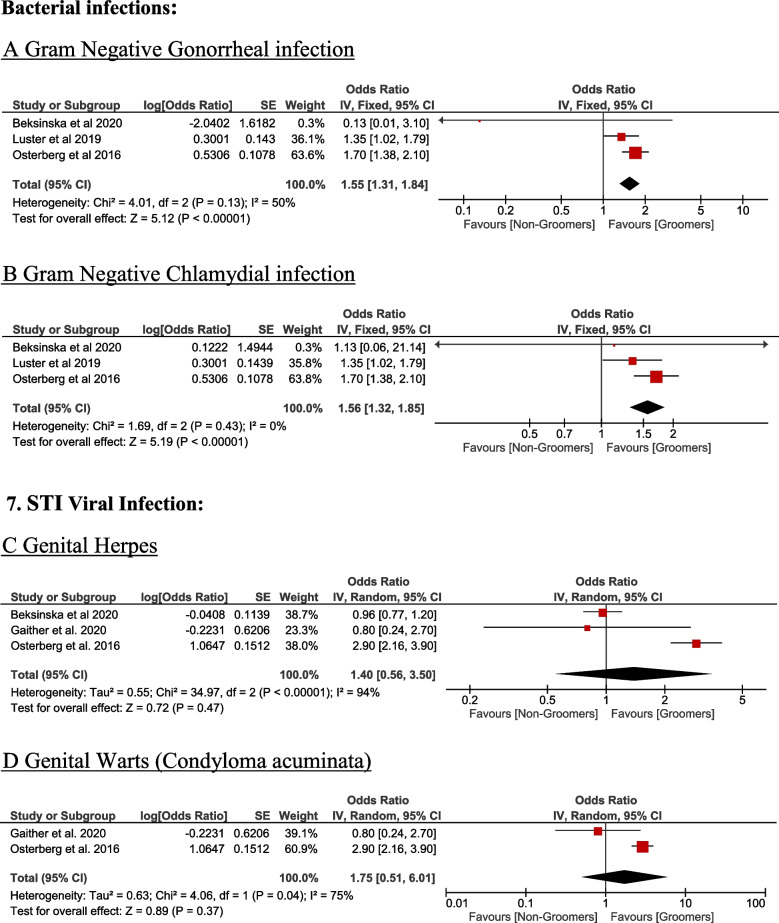
STI related to grooming practice; Bacterial infections. **A** Gram Negative Gonorrheal infection. **B** Gram Negative Chlamydial infection. Viral Infection. **C** Genital herpes. **D** Genital warts (Condyloma acuminata)

Pubic hair grooming in the female population is significantly associated with a higher odds of having gram-negative gonorrheal infection (OR = 1.55, 95% CI [1.31, 1.84], *P* < 0.001) and chlamydial infection (OR = 1.56, 95% CI [1.32, 1.85], *P* < 0.001). There was no significant difference between groomer and non-groomer women regarding viral infections such as genital herpes (OR = 1.40, 95% CI [0.56, 3.50], *P* = 0.47) and Condyloma acuminata (OR = 1.75, 95% CI [0.51, 6.01], *P* = 0.37) as shown in Fig. 7.

## Discussion

### Significance of the study

Today, body remodeling has become widespread and socially desirable, particularly in the context of pubic hair grooming. A large representative sample of women from diverse ethnicities and age groups revealed a higher prevalence of pubic hair grooming, with the majority of participants of reproductive age and white European women. Our objective is to examine the potential association between pubic hair grooming practices and sexual health, including aspects such as women's satisfaction, the occurrence of STIs, and the underlying motivations driving this grooming practice.

### Summary of findings

While this meta-analysis and systematic review included a relatively large number of studies and participants, the results of individual studies exhibit inconsistency. Included studies only partially answered our review question.

The findings of this review indicate that women's pubic hair grooming activities are more variable than commonly reported in individual studies. There was higher prevalence of pubic hair grooming among female groomers compared to non-groomers, and among white women, compared to black women. Pubic hair is more frequently groomed completely rather than partially. The most common grooming method was shaving with a non-electric razor. The most prevalent side effect of grooming was genital itching followed by genital pain and burning, genital rash, genital folliculitis and lastly genital allergy.

In the female population, Pubic hair grooming is associated significantly with a higher odd of having gram-negative gonorrheal and chlamydial infection, while there was no difference between groomer and non-groomer women regarding viral infection such as genital herpes and condyloma acuminate.

There were no differences in women’s satisfaction between the groomers and non-groomers.

### Explanation of the finding

There has been a transition in the prevalence of pubic hair grooming by women in past few years, The data collected in this review found that pubic hair grooming is a widespread practice among women, consistent with research conducted in the United Kingdom, where 86% of women aged 16 years had engaged in pubic hair grooming at a certain point in their lives [17]. In a survey of more than 2,000 women aged between 18–68 years in the United States, 80% had engaged in some form of pubic hair grooming in the prior month [18]. Also several studies have explored pubic hair removal attitudes and behaviors among diverse populations of reproductive-age women. In Italy, a study aimed to elucidate such attitudes and behaviors, revealing a prevalent popularity of pubic hair removal among the participants [19]. Similarly, a study conducted in Brazil focused on describing pubic hair removal preferences among Brazilian women, with a substantial majority (64.3%) expressing a preference for complete removal of female pubic hair [20]. In New Zealand, a study involving 584 participants reported that nearly half (48.9%) of all female respondents engaged in the removal of most or all of their pubic hair [21]. Furthermore, a study conducted in Africa, encompassing 1218 women; shed light on pubic hair grooming practices, with 58.2% of the participants, as reported by 705 women, engaging in such practices [22]. Moreover, a survey conducted on 400 Saudi women indicated that all women reported removing their pubic hair, with initiation of this practice occurring at an earlier age averaging at 13.5 years [10]. Furthermore, in another survey involving 61 Turkish women, the study revealed that a vast majority of Turkish Cypriot women regularly engaged in pubic hair removal [9].

Fifty percent of groomers choose to remove all of their pubic hair. This finding is consistent with a Canadian survey that reported 30% of women aged between 16 and 50 years as complete groomers of their pubic hair [23]. According to a New Zealand survey, 26% of women between the ages of 18 and 35 years engage in complete grooming, while an additional 25% groom the majority of their pubic hair [21].

The results illustrate the significance of ethnicity in determining the likelihood of pubic hair grooming, consistent with earlier studies [6]. Upon analysis, we identified that 80.3% of groomers were of white ethnicity, exceeding the proportion observed among black women; Aligning with previous studies that highlight a higher prevalence of grooming practices among white women compared to other racial disparities [3, 4, 6, 18, 24]. This trend may be elucidated by the observation that Black women appear to express greater satisfaction with their natural pubic condition than their White counterparts, contributing to their lower likelihood of engaging in recent pubic hair grooming practices [25], moreover limited scholarly attention has been directed toward investigating the dynamics of pubic hair removal within a demographically diverse cohort of women [6].

Similar to other reported studies, [6, 18, 26], the findings of this analysis reveal that shaving using non-electric razors is the most commonly used for pubic hair grooming activity among women, representing approximately 69.3% of all pubic hair grooming methods. This high prevalence may be explained as mass availability of razors, cheap, its resemblance to widespread behavior of shaving legs and underarms, and the fact that its rapid removal result in more repeated grooming practice and more practical as it can be conducted in home as opposed to in a cosmetic center, and so is typical with waxing [26]. However, in the Italian population, waxing is the preferred and the most common method for grooming [19].

In this review, we explore the motivation behind grooming practice and the reasons for their prevalence among women. The most commonly reported motivations were hygiene and beauty aligning with a Canadian study where the author investigated motivation behind pubic hair grooming practice, and reported that aesthetics of bikinis, beauty, femininity, hygiene, and comfort were the main drivers [23]. This is particularly pertinent for women who perceive pubic hair grooming as a symbol or marker defining the identity of their genital region [27].

Although previous research suggested that genital satisfaction is a strong determinant of pubic hair grooming frequency [18], we found no difference in genital satisfaction between groomers and non-groomers. This aligns with a study reporting that women who prefer limited pubic hair grooming are more comfortable with their genitals appearance than those who participate in more comprehensive grooming practice [25]. While the presence of pubic hair may protect women's skin from irritation, in contrast, its absence may cause irritation resulting in loss of protection against certain irritant (e.g., sex related friction or tightly fitting clothes) [26]. In a study conducted in the United States, 60% of women who groomed their pubic hair stated at least one minor grooming symptom, the most typically stated was abrasion and ingrown hairs [24]. In contrast, our review found that 26.9% of the most common retrieved complications were genital itching, genital rash, genital folliculitis, genital allergy, and genital pain and burning. While some of these complications may be minimal and only persist for a short time, they may cause epidermal microtears, potentially increasing susceptibility to infections during skin-to-skin contact [22]. This may increase the risk of STIs, notably cutaneous, viral STIs [1]. This align with our analysis, groomers were significantly associated with higher odds of having a gram-negative gonorrheal infection and chlamydial infection than non-groomers. Which in line with what it was believed that grooming pubic hair could increase the probability of complications such as STIs infection [3]. Another study identified a higher prevalence of reported STIs among individuals who engaged in grooming compared to those who did not. This positive association was consistent across various STI categories; however, the nature of these associations exhibited variability based on specific grooming practices and the type of STI under consideration [1]. While we found no difference between groomer and non-groomer women regarding viral infection such as genital herpes and condyloma acuminate, which in line with Beksinska et al., 2020, which found no difference between groomers and non- groomers in risk to acquiring HSV2 [22]. In contrast, Desruelles et al., 2013, shows there is an increased risk of condyloma acuminate infection with grooming [12]. Also Osterberg et al., 2017, found that frequent and extreme groomers were associated with high risk to infection to cutaneous STIs as molluscum contagiosum, and explained this association due to the fact that the act of grooming with razors or shavers induces microtears in the epidermis, potentially facilitating the penetration of bacterial or viral STIs such as molluscum contagiosum [1]. However, grooming is possibly confounded by many factors that may lead to increased risk of STIs. For example, grooming procedures may increase the likelihood of pubic area injuries, resulting in epidermal micro-tears, increasing vulnerability to infections, particularly cutaneous, viral STIs [1, 12, 22], STIs and complications were self-reported in many of this research which limits reliability [1, 3], The observed results of many of studies that report grooming practice and STIs might be susceptible to recall bias if participants were predisposed to report their grooming experiences [1, 3, 22, 24]. The communal utilization of grooming tools has the potential to facilitate the transmission of STIs, establishing a positive association between grooming practices and STI risk. Notably, a documented case highlights HIV transmission among siblings who shared a razor blade [1, 28]. Nevertheless, the transmission of HIV through non-sexual and non-needle sharing household contacts of individuals with HIV is exceptionally infrequent [1, 29]. Many research conducted has indicated an association between pubic hair removal and heightened sexual activity or having a sexual partner [1, 18, 22, 26, 30]. As individuals who practice grooming may exhibit a higher propensity for engaging in risky sexual behaviors compared to non-groomers.The presence of residual confounding, such as unmeasured sexual behavior practices, self-reporting biases, and potential biases in recalling STIs, may contribute to these associations [1]. In our analysis we didn’t find enough studies to explore protective effect of grooming practice and drop of pubic lice, however previous study showed the greater frequency of grooming has resulted in a drop in pubic lice [31, 32], this form of protective association aligns with findings from a previous study, wherein it was attributed to the removal of hairs, creating an environment less conducive for the hatching of louse eggs [31]. Additionally research studies from the United States, Australia, and Brazil revealed that pubic hair grooming was related to sexual behavior, such as having an intimate sexual spouse, examining one's own genitals within the past month, engaging in cunnilingus over the previous month, and experiencing a more positive genital self-image or engaging in specific sexual practices [18, 20, 30], Such as vaginal fingering and finger–clitoral stimulation, having a casual sex partner, utilizing vaginal hygiene products, and applying cream to the genitals. Additionally, there was a marginal association between hair removal and a longer duration of vaginal penetration [26], therefore removal of pubic hair emerges as a significant facet of expressing one's sexuality and engaging in sexual activity, presenting an intriguing psychosexual foundation that remains incompletely explored in the field of sexual medicine [11]. Therefore, during routine visits, clinicians should discuss potential expected issues and offer safe methods for conducting pubic hair grooming practice, as well as discuss genital health and hygiene.

### Implications of these findings in practice

Based on our current findings from this review, we can gain a better understanding of female pubic hair grooming in the European environment. In combination with clinical data about pubic hair grooming's benefits and risks, it can assist health care providers to detect groups expected to be affected the most by pubic hair grooming-related health outcomes, especially in adolescence and childbearing age. When faced with a pubic hair grooming problem, educate these patients, align reasons behind grooming practices, and develop guidelines that will result in better health outcomes. According to our findings, complete grooming is more common among white women. In addition, we found statistically significant difference in Gonorrhea and Chlamydia infections between groomers and non-groomers.

For a better understanding, more research using qualitative and quantitative methodologies will be needed to study psychological, social, and sexual factors. This includes investigating grooming methods, the frequency of grooming, and health issues associated with grooming practices for both males and females.

## Strength points and limitations

There are several limitations to this exploratory study. We analyzed cross-sectional data that did not provide us with more information about motivations for pubic hair grooming, injury frequency, or in-depth questions about motivations.

Due to a lack of approved tools, the majority of the data was acquired through self-designed questionnaires. Despite adding literature to improve its validity and reliability, the use of a non-validated measure is a barrier. It is, however, important to note that the relatively large sample size obtained from this review still cannot fully compensate for the fact that this study depend on non-probability samples of cross-sectional surveys and retrospective recalls that may be highly biased. In spite of the fact that pubic hair grooming seems to be prevalent, most of research papers are confined to European countries and are not representative of other populations in Africa and the Middle East, As a result, there is a limitation in understanding the social, sexual, and behavioral aspects related to pubic hair grooming practice and associated health issues in this populations.

The reported motivation and satisfaction could be less representative as well. The study only collected data on four STIs; therefore, we are unable eliminates the chance of bidirectional causation. Furthermore, results may not be applicable among different populations with diverse geographic and racial backgrounds. However, we believe these limitations are overcome by the fact that this is the first systematic review and meta-analysis combining data from all cross-sectional studies of diverse populations and ethnicity. This review's findings shed light on the prevalence and scope of pubic hair grooming behaviors among women and how it fluctuates according to certain variables from a health promotion viewpoint. In addition, the synchronized gathering of data on health issues (e.g., Injuries, STIs) and behavioral attitude (e.g., motivation and satisfaction regarding pubic hair grooming) permitted us to better understand the social basics of pubic hair grooming practice.

## Recommendations for future research and clinical practice

Pubic hair begins to appear at the onset of puberty, and discussions about pubic hair and ﻿its grooming practices should be integrated into early health education, training, and skills programs for girls. This integration will facilitate the education and orientations of women at an early age, empowering them to protect themselves from health hazards related to improper grooming practices and promote self-hygiene [4]. Furthermore it addresses the methods by which they deal with or change or improve their genitals shape this assists doctors, health educators to cooperate together with women on these matters with better attention and efficiency. Therefore, continued research in this area will be necessary, allowing clinicians to respond to their patients from an evidence-based perspective.

Finally, future research should explore the full picture of differences in pubic hair grooming practice, methods, benefits, and health hazards (e.g., STIs) among women in different geographic scopes and races to enhance awareness of pubic hair grooming practice.

## Conclusion

Pubic hair grooming is a normative practice with a clear relational and sexual character. Our findings support the idea that pubic hair grooming malpractice is considered a risk factor for STIs which aligns with previous studies regarding the health hazards of pubic hair grooming and the racial disparities in this practice. While our study contributes significant insights into grooming practices and their societal influences, it is essential to acknowledge certain limitations that warrant consideration, one notable limitation is the skewness of the data towards a predominantly European focus. That may have inadvertently led to an underrepresentation of grooming practices in other cultural contexts. Consequently, the generalizability of our findings to a more diverse global population may be constrained. Furthermore, there is a crucial need to heighten the awareness of women regarding the safe practice of pubic hair grooming with clarification of hazards and benefits.

### Supplementary Information

**Additional file 1.** Pubic hair removal. (Search strategy).

**Additional file 2.**

## Data Availability

All data generated or analyzed during this study are included in this published article [and its supplementary information files].
