# Supplementary material for: Effects of pubic hair grooming on women’s sexual health: a systematic review and meta-analysis
Source: BMC Womens Health. 2024 Mar 11;24:171. doi: 10.1186/s12905-024-02951-1 (PMC10926629; doi:10.1186/s12905-024-02951-1)
Supplement: Supplementary file 1 — Additional file 1. Pubic hair removal. (Search strategy). [file 12905_2024_2951_MOESM1_ESM.docx]

Pubic Hair Removal

(Search Strategy)

PubMed Search:

(Pubic hair remov* OR (pubic hairless*) OR (genital hairless*) OR (pubic hair withdraw*) OR (pubic hair epilat*) OR (pubic hair take away) OR (genital shaving) OR (perineal shaving) OR (pudendal shaving) OR (private hair removal) OR (bikini hair removal) OR (pubic hair depilate*) OR (pubic hair wax*) OR (pubic hair Sugar*) OR (pubic hair pluk*) OR (pubic hair shed*) OR (pubic hair laser*) OR (pubic hair electrolysing*) OR (mons pubic hair ) OR (pubic hair trim*) OR (pubic hair preen*) OR (pubic hair follicle*)

Result: 662

Cochrane Search:

(pubic hair remov*)OR (pubic hairless*) OR (genital hairless*) OR (pubic hair withdraw*) OR (pubic hair epilat*) OR (pubic hair take away*) OR (genital shaving*) OR (perineal shaving*) OR (pudendal shaving*) OR (private hair removal*) OR (bikini hair removal*) OR (pubic hair depilate*) OR (pubic hair wax*) OR (pubic hair Sugar*) OR (pubic hair pluking*) OR (pubic hair shed*) OR (pubic hair laser*) OR (pubic hair electrolys*) OR (mons pubic hair* ) OR (pubic hair trim*) OR (pubic hair preen*) OR (pubic hair follicle*)

Result: 45

Scopus:

(pubic AND hair AND remov* ) OR ( pubic AND hair AND shav* ) OR ( genital AND hairless ) OR ( pubic AND hair AND groom* ) OR ( pubic AND hair AND epilat* ) OR ( genital AND shav* ) OR ( perineal AND shaving ) OR ( private AND hair AND remov* ) OR ( pubic AND hair AND laser* ) OR ( pubic AND hair AND electroly* ) OR ( pubic AND hair AND follicle* ) OR ( pudendal AND shaving ) OR ( bikini AND hair AND removal ) OR ( pubic AND hair AND trim* ) OR ( mons AND pubic AND hair ) OR ( pubic AND hair AND wax* ) OR ( pubic AND hair AND sugar)

Results: 715

Web of Science

(pubic AND hair AND remov* ) OR ( pubic AND hair AND shav* ) OR ( genital AND hairless ) OR ( pubic AND hair AND groom* ) OR ( pubic AND hair AND epilat* ) OR ( genital AND shav* ) OR ( perineal AND shaving ) OR ( private AND hair AND remov* ) OR ( pubic AND hair AND laser* ) OR ( pubic AND hair AND electroly* ) OR ( pubic AND hair AND follicle* ) OR ( pudendal AND shaving ) OR ( bikini AND hair AND removal ) OR ( pubic AND hair AND trim* ) OR ( mons AND pubic AND hair ) OR ( pubic AND hair AND wax* ) OR ( pubic AND hair AND sugar)

Results: 465

OVID

(pubic AND hair AND remov* ) OR ( pubic AND hair AND shav* ) OR ( genital AND hairless ) OR ( pubic AND hair AND groom* ) OR ( pubic AND hair AND epilat* ) OR ( genital AND shav* ) OR ( perineal AND shaving ) OR ( private AND hair AND remov* ) OR ( pubic AND hair AND laser* ) OR ( pubic AND hair AND electroly* ) OR ( pubic AND hair AND follicle* ) OR ( pudendal AND shaving ) OR ( bikini AND hair AND removal ) OR ( pubic AND hair AND trim* ) OR ( mons AND pubic AND hair ) OR ( pubic AND hair AND wax* ) OR ( pubic AND hair AND sugar)

Results: 1596
